# Quantum-dot micropillar lasers subject to coherent time-delayed optical feedback from a short external cavity

**DOI:** 10.1038/s41598-018-36599-3

**Published:** 2019-01-24

**Authors:** Steffen Holzinger, Christian Schneider, Sven Höfling, Xavier Porte, Stephan Reitzenstein

**Affiliations:** 1 Institut für Festkörperphysik, Quantum Devices Group, Technische Universität Berlin, Hardenbergstraße 36, 10623 Berlin, Germany; 2 Technische Physik, Universität Würzburg, Am Hubland, 97074 Würzburg, Germany; 3 SUPA, School of Physics and Astronomy, University of St Andrews, St Andrews, KY16 9SS United Kingdom

## Abstract

We investigate the mode-switching dynamics of an electrically driven bimodal quantum-dot micropillar laser when subject to delayed coherent optical feedback from a short external cavity. We experimentally characterize how the external cavity length, being on the same order than the microlaser’s coherence length, influences the spectral and dynamical properties of the micropillar laser. Moreover, we determine the relaxation oscillation frequency of the micropillar by superimposing optical pulse injection to a dc current. It is found that the optical pulse can be used to disturb the feedback-coupled laser within one roundtrip time in such a way that it reaches the same output power as if no feedback was present. Our results do not only expand the understanding of microlasers when subject to optical feedback from short external cavities, but pave the way towards tailoring the properties of this key nanophotonic system for studies in the quantum regime of self-feedback and its implementation to integrated photonic circuits.

## Introduction

Quantum-dot micropillar lasers constitute a flexible and mature testbed for exploring the exciting physics at the crossroads between quantum nanophotonics and nonlinear laser dynamics. From a device physics perspective, the small dimensions and strong interaction between optical field and cavity cause micropillar lasers to operate in the regime of cavity quantum electrodynamics (cQED), where single-emitter light-matter interaction and high spontaneous emission noise become prominent^1^ and interesting effects such as superradiance can be observed^2^. Micropillars with a low number of quantum-dots (QD) as gain medium are profusely used in the context of quantum optics. Here effects like strong coupling have been demonstrated^3^. Moreover, such systems can be used as triggered sources of single indistinguishable photons^4–7^. When the system is pumped stronger or when the QD density is increased, the transition to lasing in QD micropillars can be studied^8^ close to the regime of thresholdless lasing^9,10^. With respect to nonlinear dynamics microlasers exhibit noise-enhanced nonlinear phenomena such as partial locking^11^, spontaneous symmetry breaking^12^ and extreme events^13^.

It is well known that complex nonlinear dynamics and chaos can be triggered in semiconductor lasers via the addition of delayed feedback or optical coupling^14,15^. In particular, the nonlinear dynamics of feedback-coupled lasers has been extensively studied in a wide parameter range^16,17^ paving the way for applications that range from random number generation^18^ to chaos communications^19^. However, there are still many interesting open questions when addressing delayed feedback, especially for microlasers operating at ultra-low optical powers in the limit of few photons in the cavity approaching the transition between classical physics and quantum physics. While early works already spotted feedback-induced complex dynamics in this regime^20,21^, recent publications highlight the possibility to tailor the mode-switching dynamics in this system via delayed optical feedback^22,23^. However, all these works were based on cavity lengths much longer than both the coherence time of the laser and the relaxation oscillation period, leaving open the question of how phase sensitivity affects the dynamics. Furthermore, a fundamental dynamical time scale in lasers like the relaxation oscillation frequency and its possible dependence on optical feedback has not yet been studied in microlasers. In the regime of very short cavities complex dynamics such as regular pulse packages can be observed with timescales that are dominated by the external cavity roundtrip time rather than by the relaxation oscillations as in the long cavity regime^24^. These very short cavities are naturally present in integrated photonic circuits^25^ and can even be tailored to enable switching between the aforementioned dynamic regimes^26^. Moreover, integrated photonic circuits allow direct generation of broadband chaos^27^ which can be exploited for fast physical random number generation^28^. Interestingly, in the single-emitter single-photon quantum regime it has been predicted that coherent feedback can enhance the fidelity of polarization entangled photon pairs emitted by a single QD^29^.

Here, we study the effects of feedback from a short external cavity on the dynamics of a QD micropillar laser. We explore in detail how feedback influences the optical spectrum and the second-order autocorrelation function. By changing the pump current and the external cavity length we can study the interplay of the system’s time scales, namely the coherence time, the external cavity length and relaxation oscillation frequency. Here the microlaser’s relaxation oscillation frequency is determined by injecting ps-length optical pulses and measuring its response. Moreover, we observe the dynamical turn-on of the feedback state in the microlaser. Interestingly, the optical pulse injection appears to reset the feedback-coupled system to its solitary state, only building up the feedback-coupled conditions one external cavity round-trip after.

## Results

### Power-current and spectral characteristics

**Figure 1:**
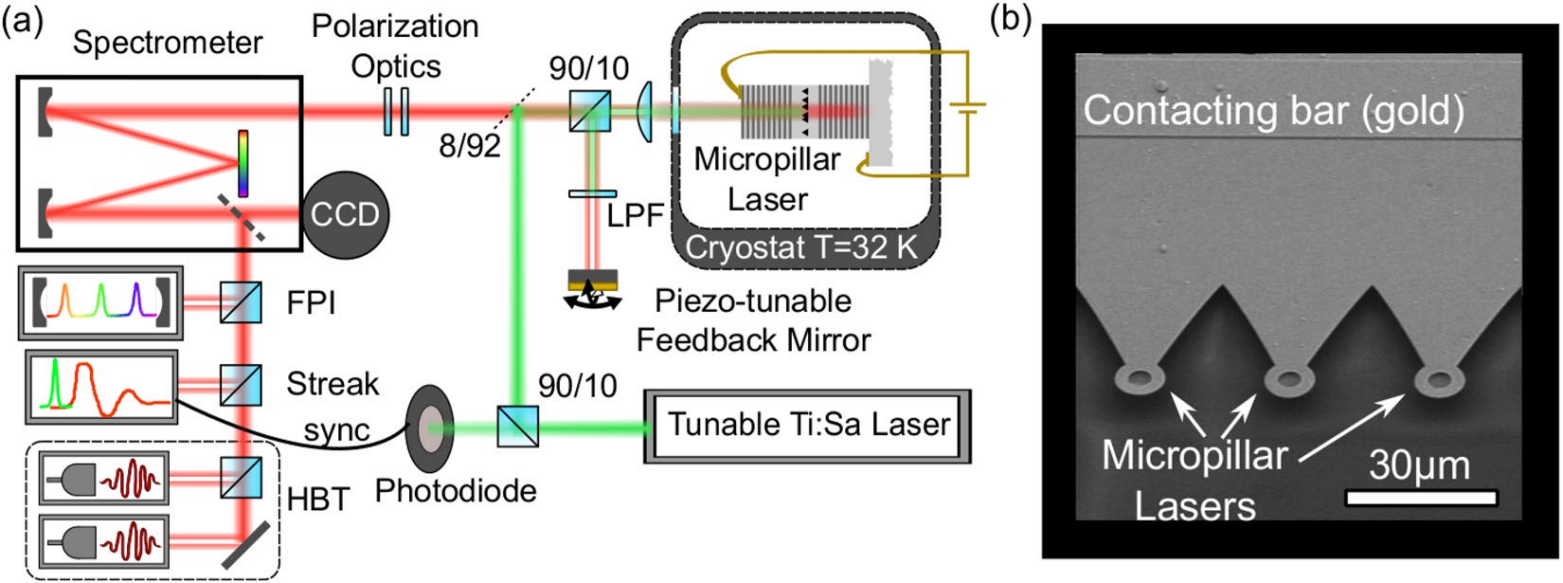
(**a**) Sketch of the micro-electroluminescence setup. The micropillar is kept at cryogenic temperatures (*T* = 32 K) inside a He-flow cryostat. The spectral properties are investigated with a grating spectrometer equipped with a CCD camera and Fabry-Perot interferometer (FPI) for increased spectral resolution. The photon statistics is characterized via two single-photon counting modules (SPCMs) which form a Hanbury Brown and Twiss (HBT) setup. A streak camera in synchronscan mode records the temporal response of the microlaser to an injected pulse from a tunable Ti:Sa laser. A long pass filter (LPF) in the external cavity suppresses feedback from the Ti:Sa pulse. (**b**) Scanning electron microscope image of the used mircopillar structures.

Firstly, we present the effects of delayed optical feedback from a short cavity on the input-output characteristics and the optical spectrum of a QD micropillar laser. We perform micro-electroluminescence (μEL) experiments on a QD micropillar with a diameter of 5 μm as shown in Fig. 1. (Please see Methods section for more details on the experimental setup and device technology.) In such electrically driven QD-micropillars, lasing emission originates from the Gaussian fundamental mode, which exhibits a two-fold degeneracy. This degeneracy is usually lifted due to slight structural asymmetries of the micropillar leading to gain competition between the resulting modes^30^. We label the mode winning the gain competition above lasing threshold *strong mode* (SM) while the other mode is called *weak mode* (WM). Higher order transverse modes as typically observed in VCSELs^31^ can be neglected here as the side mode suppression ratio (SMSR) for these respective modes exceeds 25 dB above laser threshold.

**Figure 2:**
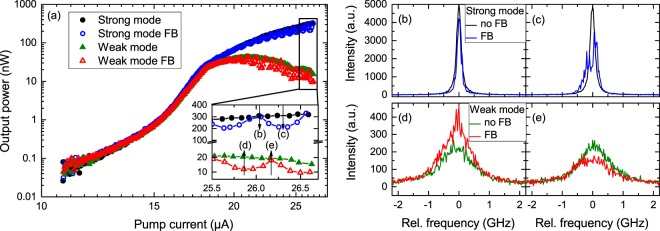
(**a**) Input-output characteristics of the investigated microlaser. The inset highlights the output power modulation caused by the short cavity feedback. Arrows indicate the four pump conditions of the right panels (**b** to **e**). High resolution FPI spectra of the strong mode being (**b**) in phase and (**c**) out of phase with the ECMs of the external cavity. Accordingly, (**d**,**e**) show respective cases of the weak mode.

Figure 2(a) depicts the input-output characteristics of the investigated microlaser emitting at a wavelength of 900.1 nm. From the measured linewidth at inversion we extract a quality (*Q*) factor of ~21000. The strong mode wins the gain competition at the lasing threshold ($I\simeq 16\,{\rm{\mu }}A$) resulting in a shallow s-shape when presented on a double-logarithmic scale. From fitting of the input-output characteristics with a semi-classical rate equations model^22,32^ we determine a spontaneous emission factor of β = 4 · 10^−3^, which is considerably higher than typically observed in classical semiconductor lasers (β ~ 10^−4^ to 10^−6^). The rate equations model as well as the full set of fitting parameters can be found in^32^ as the investigated micropillar is identical to pillar 2 in the mentioned publication. In contrast, the weak mode intensity increases sublinearly around threshold and finally even decreases for pump currents *I* > 20 μA.

In the inset of Fig. 2(a) we observe a modulation of the emission intensity resulting from the application of optical feedback from a short cavity. This can be explained by the phase-coherent interaction between the solitary microlaser mode the external cavity modes (ECMs). The latter ones are distributed in a comb of equidistant modes with a separation of ${\rm{\Delta }}{\nu }_{ECM}=\frac{1}{{\tau }_{ext}}=1.03\,{\rm{GHz}}$. By increasing the pump current, the strong mode shifts first to higher energies (as increasing carrier concentration leads to an increase in refractive index) and then to lower energies due to sample heating for high pump current. Thus, crossing several ECMs in the process. Each crossing is related to one period of intensity modulation in the input-output. The external cavity roundtrip time is as short as ${\tau }_{ext}=0.97\,{\rm{ns}}$. Directly above lasing threshold, when both modes split in intensity ($I\simeq 6\,{\rm{\mu }}A$), the coherence time of both modes ${\tau }_{coh}=0.32\,{\rm{ns}}$ is still smaller than *τ*_*ext*_. Further increasing the pump current only leads to a minor increase of the weak mode coherence time to ${\tau }_{coh,WM}=0.54\,{\rm{ns}}$, while the strong mode coherence time increases to ${\tau }_{coh,SM}=1.90\,{\rm{ns}}$ which is greater than *τ*_*ext*_. This means that the intensity modulation is visible in presence of delayed feedback if the condition ${\tau }_{ext}\lesssim 3{\tau }_{coh}$ is fulfilled. A more detailed study of the output power amplitude modulation will be discussed later.

Figure 2(b–e) depict high resolution Fabry-Perot interferometer (FPI) spectra taken for the strong mode and the weak mode when these modes are in and out of resonance with an ECM. The solitary spectra are shown in different colors (black, green) for comparison. Being in resonance with an ECM (cases (b) and (d)) leads to a reduction in linewidth and thus an increase in coherence time *τ*_*coh*_ from 1.86 ns to 2.74 ns and from 254 ps to 318 ps, respectively. For the strong mode the FWHM is reduced to about 116 MHz, close to the resolution limit of the FPI ($\sim 100\,{\rm{MHz}}$) when it is in phase with an ECM. The linewidth reduction might thus be more significant than we can resolve with the used FPI. When the fundamental mode is out of resonance with an ECM (cases (c) and (e)) the spectrum broadens because of destructive phase-interference between the lasing mode and the external cavity modes^33^. The SM is between two ECMs resulting in a phase instability which also explains the lowered average fitted intensity in the input-output characteristics recorded on the CCD.

### Second-order autocorrelation and phase dependence of the dynamics

In this section, the second-order autocorrelation function *g*^(2)^(*τ*) is used as a sensitive measurement for identifying the dynamics in micropillar lasers under phase-coherent feedback. In this context we would like to note that photodetector efficiencies in the near infrared spectral range are insufficient to capture the dynamics at nW light levels with full temporal resolution, therefore intensity time traces with acceptable bandwidth are experimentally not achievable. To detect the intensity fluctuations of the microlaser emission we use single-photon counting modules (SPCM) that have high sensitivity and fast response times. We use two SPCM set up in a Hanbury Brown and Twiss configuration to extract *g*^(2)^(*τ*) and to explore the underlying dynamics^20^.

Previous studies on similar micropillars have shown that the modes exhibit switching dynamics with the weak mode emitting mainly in a thermal state with short switching periods where it emits in a lasing state via emission of multiphoton pulses (vice versa for the strong mode)^22^. By measuring *g*^(2)^(*τ*) we will study the characteristic switching timescales and the stability of the modes to obtain a detailed understanding of they system in the short external cavity regime.

**Figure 3:**
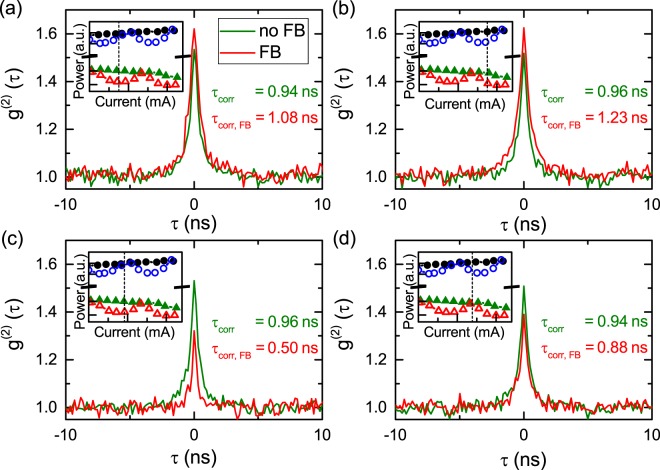
Autocorrelation measurements for various phase conditions of weak mode. Insets show the range of interest along the input-output curve with a dashed line indicating the used pump current from which one can deduce the feedback phase of both modes. (**a**) Weak mode is out of phase while strong mode is not in phase. (**b**) Both modes are not in phase. (**c**) Strong mode is in phase while weak mode is not. (**d**) Weak mode is in phase while strong mode is not.

Figure 3 shows the second-order autocorrelation function of the weak mode for four different currents ranging from 25.9 to 26.2 μA. Only *g*^(2)^(*τ*) of the weak mode is shown because the strong mode exhibits *g*^(2)^(*τ*) ≊ 1 for most pump current conditions. This behavior is typical for micropillars in which the strong mode dominates the gain competition^20,22^. The pronounced intensity divergence between SM and WM in this pillar prevent us to see any competing dynamics that would actually result in bunching and revival peaks in both modes^23^. The weak mode without feedback exhibits bunching at zero time delay of ${g}^{\mathrm{(2)}}\mathrm{(0)}=1.53\pm 0.02$ in this pump regime. Taking into account the correlation time ${\tau }_{corr}=\mathrm{(0.95}\pm \mathrm{0.05)}\,{\rm{ns}}$ (which is the time constant extracted from the exponential decay of the *g*^(2)^-function) being significantly greater than the coherence time ${\tau }_{coh}=\mathrm{(0.26}\pm \mathrm{0.01)}\,{\rm{ns}}$, this bunching cannot be attributed to thermal emission only. Cross-correlation measurements in which the strong mode and weak mode are detected on one SPCM each show ${g}^{\mathrm{(2)}}{\mathrm{(0)}}_{SM-WM} < 1$, proving the anti-correlation of both modes^23^.

The external cavity length for this experiment is chosen such that both modes have different feedback-phase conditions for the investigated current. Figure 3(a) depicts the case where the weak mode is out of phase while the strong mode is not in phase, whereas in panel (b) both modes are not in phase. Both cases show an increase in bunching of the weak mode that can be explained by an increased rate of multiphoton pulses of the weak mode. Correlation times increase accordingly to ${\tau }_{corr}=\mathrm{(1.08}\pm \mathrm{0.05)}\,{\rm{ns}}$ and ${\tau }_{corr}=\mathrm{(1.23}\pm \mathrm{0.05)}\,{\rm{ns}}$. Panels (c) and (d) exhibit the case where either the strong or the weak mode are in phase while its counterpart is out of phase. Both scenarios lead to a significant decrease in bunching as in both cases one mode is stabilized, which suppresses the switching dynamics. The correlation times decrease to ${\tau }_{corr}=\mathrm{(0.50}\pm \mathrm{0.05)}\,{\rm{ns}}$ and ${\tau }_{corr}=\mathrm{(0.88}\pm \mathrm{0.05)}\,{\rm{ns}}$. Thus, one can conclude that switching dynamics are highly sensitive to the feedback phase in the short cavity regime. Moreover, it is clear that for arbitrary phase relations of the feedback phase both modes have to be considered when trying to enhance or suppress the dynamics. We found that an increase in bunching and the switching time scale only occurs when none of the solitary modes is in phase with an ECM (Fig. 2(a,b)), whereas a decrease of those parameters will occur if at least one mode is in phase. The same tuning effect can be achieved by changing the cavity length with e.g. a mirror mounted on a piezo translator and a loudspeaker^34^ but here we focus on pump current tuning as it is naturally accessible in microlasers and integrated photonics.

### Phase sensitivity

The main characteristic of the short cavity regime, in contrast to the long cavity regime, is that the optical spectrum and dynamics are sensitive to the optical feedback phase. Here, we investigate the boundaries between both regimes and we aim at identifying a relation to the coherence time of the laser by varying the external cavity length. We define the output power amplitude modulation (AM) as the amplitude of the sinusoidal fit of the modulated output intensity for both SM and WM.

**Figure 4:**
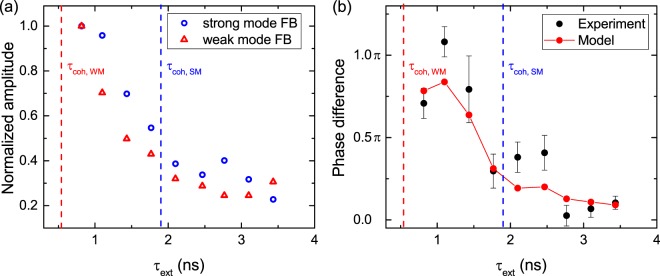
Cavity length series. (**a**) Amplitude modulation of both strong and weak mode as a function of the cavity roundtrip time. (**b**) Phase difference of the amplitude modulation of both modes shown as black squares. An empirical model is depicted in red. The coherence times of both modes are indicated by dashed blue and red lines, respectively.

Figure 4(a) depicts the AM as a function of the external cavity roundtrip time. One can identify that the exponential decay of the AM resembles the fringe contrast function of a Michelson interferometer, which is commonly used to determine the coherence time of lasers. Analogously, the AM does not drop immediately to zero for ${\tau }_{ext} > {\tau }_{coh}$ but follows an exponential decay. We want to note that one needs to increase the sampling through the input-output curves to properly resolve the modulation when further increasing the external cavity roundtrip time. For example, at least 25 data points per μA are needed to resolve this modulation for an external cavity roundtrip time of ${\tau }_{ext}=3.4\,{\rm{ns}}$. Taking too coarse steps in pump current will therefore lead to aliasing which is masking the modulation. Interestingly, the AM decay shows similar behavior for both modes even though their coherence times differ significantly (${\tau }_{coh,WM}=0.54\,{\rm{ns}} < {\tau }_{coh,SM}=1.90\,{\rm{ns}}$). This interesting observation can be attributed to on-off switching of the modes as the multiphoton pulses of the WM have a significantly higher coherence time than detected in the averaged measurement of the optical spectrum.

Figure 4(b) shows the phase difference of the amplitude modulation of strong and weak modes when modifying the external cavity length. The linewidth of the solitary strong mode is ${\rm{\Delta }}{\nu }_{SM}=174\,{\rm{MHz}}$ while the weak mode exhibits a linewidth of ${\rm{\Delta }}{\nu }_{WM}=1.21\,{\rm{GHz}}$. For the investigated range of external cavity lengths ${\tau }_{ext,min}=0.82\,{\rm{ns}}$ to ${\tau }_{ext,max}=3.4\,{\rm{ns}}$ the strong mode can only interact with one ECM within its linewidth while the weak mode couples to multiple modes as ${\rm{\Delta }}{\nu }_{ECM}$ ranging from 1.22 GHz to 294 MHz. Thus, we developed an empirical equation to model the feedback phase difference ${{\rm{\Delta }}{\rm{\Phi }}}_{S-W}$ between strong and weak modes:

1 $${{\rm{\Delta }}{\rm{\Phi }}}_{S-W}=mod[2\pi \,\ast \,\frac{1}{n}\sum _{j=1}^{n}\,mod[{\rm{\Delta }}{\nu }_{S-W}({I}_{j}),{\rm{\Delta }}{\nu }_{ECM}],2\pi ]\cdot \frac{1}{N},$$

where ${\rm{\Delta }}{\nu }_{S-W}({I}_{j})$ is the current dependent spectral detuning of both modes above threshold and N is the amount of ECMs that fit into the full-width half maximum linewidth of the weak mode. The value of the phase difference is reduced the more ECMs are within the linewidth of the weak mode resulting in an anti-phase relation for short cavities. For ${\tau }_{ext}\gg {\tau }_{coh}$ the phase difference approaches zero and can be set to a constant value, which is typically done in numerical simulations^35^. Thus, this effect can be exploited to precisely control the phase difference of two modes. It is underlined that the model is only used for cavity lengths where the current dependent spectral detuning is known from the measurement.

### Relaxation oscillation measurements

Feedback dynamics are influenced by the interplay of the microlaser time scales, in particular the relaxation oscillation frequency ${f}_{RO}=\frac{1}{{\tau }_{RO}}$ and external cavity round trip time *τ*_*ext*_^15,17^. Thus, it is crucial to determine the ratio of these time scales. For this purpose we check how *f*_*RO*_ are affected by the short cavity feedback. Established methods for determining *f*_*RO*_ such as acquiring the relative intensity noise spectrum^36^ (e.g. with a photo diode and an electrical spectrum analyzer) again fail at the light levels well below 1 *μ*W in our experiment. To circumvent this issue we developed an approach where we inject picosecond optical pulses from a Ti:Sa laser to off-resonantly excite the electrically dc-biased microlaser (similar to^37^) and to measure its optical response on a streak camera (see Methods for more details). The injected optical pulse is effectively used as a short signal modulation that triggers a relaxation oscillation. Thus, using this superposition of DC bias and optical pulses we can address the exact in phase and out of phase conditions from previous measurements.

**Figure 5:**
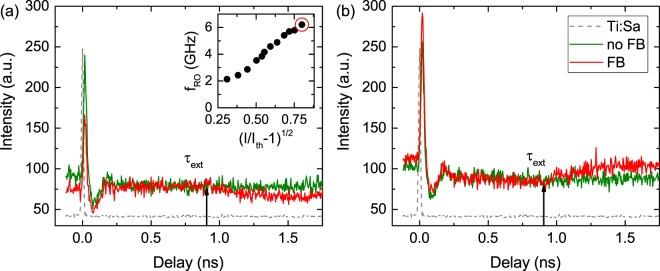
Streak camera measurements depicting the weak mode without and with feedback for both (**a**) out of phase (see Fig. 3(a,b)) and (**b**) in phase (see Fig. 3(d)) conditions. As a time reference the Ti:Sa pulse is shown as a gray dashed line. Vertical black arrows indicate the external cavity roundtrip time. The inset highlights the evaluated relaxation oscillation frequency as a function of the square root pump current above threshold. The red circle marks the measurement shown in panel (a).

Figure 5 depicts the streak camera time trace for the WM being out of phase (a) and in phase (b) with the ECMs. We can observe that optical feedback has no significant influence on the relaxation oscillation frequency or its damping. This can be explained by the strong damping of the relaxation oscillations previously reported for QD lasers^38,39^, which is proportional to the critical feedback strength needed to destabilize the cw laser operation^40–42^. This behavior is preserved in the microlasers making them an ideal candidate for lasers in applications that have to be robust against spurious reflections. The inset of Fig. 5 depicts the pump current dependence of the relaxation oscillation frequency which shows the expected square-root behavior with pump current for single-mode semiconductor lasers^43^. The deviations from this dependence for low pump currents close to threshold can be explained by the broad linewidth and low SMSR which are in contrast to the assumption that the laser spectrum can be narrowed down to a single frequency^44^. Discrepancies for higher pump currents can be attributed to sample heating, causing red shifting of the QD gain^45^. The streak camera trace shown in panel (a) is carried out with a pump current (highlighted by a red circle in the inset) equal to the measurements of Fig. 3. One can determine a relaxation oscillation frequency ${f}_{RO}=6.2\,{\rm{GHz}}$ being equal to ${\tau }_{RO}=161\,{\rm{ps}}$ which significantly smaller than the external cavity round trip times used in the experiments above. It would be interesting to study short-cavity feedback effects also on the order of *τ*_*RO*_ because this regime has been identified in semiconductor lasers to exhibit interesting dynamics which are sensitive to the external cavity roundtrip time such as regular pulse packages^24^. However, required cavity lengths of about 2 cm are not feasible in the present experimental configuration. In order to naturally access these ranges of short cavities integrated devices may provide an attractive option^26^.

While we do not see any undamping of the relaxation oscillations which would have been accompanied by revival peaks in *g*^(2)^(*τ*), we instead discover an interesting effect related to the used optical pulse. When the system is prepared in a state where feedback leads to a reduction or an increase of the output power, the optical pulse effectively resets the system to its state where no feedback is present. After one external cavity roundtrip time the system relaxes back to its stationary feedback state. Unlike topological solitons in semiconductor lasers with optical feedback, which have been suggested for coherent optical communication networks as phase bits^46^, we could exploit the feedback phase in the mentioned scheme to realize intensity bits. Furthermore, the external pulse that is resetting the stationary feedback state can be used for clock recovery. Here, the clocking bandwidth can be increased by further reducing the external cavity length. For now QD micropillar lasers offer an excellent testbed system for investigating fundamental laser physics and nonlinear dynamics. However, for applications it is desirable to move towards room temperature operation, which at the moment is only possible using long-wavelength InGaAs QDs^47,48^ or other low-dimensional gain materials^49,50^.

## Conclusions

In conclusion, we study optical feedback from a short cavity on a bimodal QD microlaser operating in the regime of cQED with sub-*μ*W output power. We analyze the change of the optical spectrum and the second-order autocorrelation function when feedback with a delay smaller than the coherence time of the laser above threshold is applied. The relative phase difference between laser cavity and external cavity is controlled by precisely varying the pump current of the microlaser. Therefore, it is possible to address both in phase and out of phase feedback configurations for each mode, thus enabling both an increase and decrease in coherence time. The former is accompanied by linewidth narrowing which leads to a stabilization of respective mode while the latter is characterized by linewidth broadening resulting in destabilization. Lastly, we characterize the relaxation oscillation frequency of the microlaser via the injection of ps-length optical pulses. Feedback neither modified *f*_*RO*_ nor its damping. On the contrary, a resetting effect of the feedback state was observed after the optical pulse, only coming back to the “stationary” feedback levels after one cavity roundtrip time. The presented findings aim to improve the understanding and tailoring of the optical spectra and nonlinear dynamics of high-*β* microlasers via external optical coupling at ultra-low light levels. Moreover, it is also relevant to applications like photonic reservoir computing^51^ and photonic integrated circuits.

## Methods

### Sample

The electrically driven micropillar lasers investigated in the experiments use a single layer of In_0.3_Ga_0.7_ As quantum dots with an area density of 5 · 10^9^ cm^2^ as a gain medium. The active layer is placed in the center of a *λ*-cavity which is formed by an upper and lower distributed Bragg reflector constituted of 23 and 27 alternating quarter wavelength thick bilayers of AlAs/GaAs, respectively. Pillar structures with a diameter of 5 μm are fabricated using electron-beam lithography and plasma etching. The sample is planarized with the dielectric benzocyclobuthene (BCB) which enables electrical contacting of the microlaser via ring-shaped gold contacts. For further details on sample fabrication see^52^.

### Experimental Setup

During all investigations the sample is mounted in a liquid-Helium flow cryostat and stabilized at a temperature of *T* = 32.00 K with ±0.01 K precision. While this temperature is chosen for spectral matching the fundamental cavity mode with the gain maximum of the QDs, lasing is found up to ~100 K for the used gain medium based on InGaAs QDs^53^. Electrical excitation is provided by an Agilent B2900 source and measurement unit. Laser emission is collimated by an aspherical lens (NA = 0.5) and spatially filtered by a pinhole. Input-output characteristics are integrated from spectra recorded with a grating spectrometer with an attached charge-coupled device camera (spectral resolution: 6.5 GHz) while high-resolution spectra are taken with scanning Fabry-Perot etalon (7.5 GHz free spectral range, 100 MHz resolution). A Hanbury-Brown and Twiss interferometric setup is formed by a 50:50 multi-mode beam splitter coupled to two Si based single photon counting modules (SPCMs) with a temporal resolution of 57 ps. The SPCMs signals are read out by a qTools qTau time-to-digital converter. A Hamamatsu universal streak camera which is synchronized to a pulsed 80 MHz Spectra-Physics Tsunami titanium-sapphire laser (Ti:Sa, 2 ps pulse length) is used to determine the relaxation oscillation frequency. The optical pulses are off-resonantly (849 nm) to the lasing mode injected into the first mininmum of the DBR mirror. The pulse intensity is chosen such that the system is brought it of its equilibrium but not leading to an effective pumping of the microlaser. A longpass filter in the external cavity ensures that the Ti:Sa pulses are not fed back into the micropillar. A grating spectrometer in front of the streak camera enables simultaneous spectrally resolved measurements of the microlaser emission as well as the Ti:Sa pulse reflected from the micropillar which is vital for determining the turn-on delay.

## Data Availability

The data of this study are available from the corresponding authors on reasonable request.
